# Elevated CO_2_ and Water Limitation Alter the Primary Metabolite Composition of Mealybug Honeydew, Reducing Parasitoid Fitness

**DOI:** 10.1021/acs.jafc.5c17227

**Published:** 2026-06-26

**Authors:** Pablo Urbaneja-Bernat, Maria Schulze-Sylvester, Angeliki Syropoulou, Caroline Müller, Rabea Schweiger, Christine Becker

**Affiliations:** † Plant Immunity and Biochemistry Group, Biology, Biochemistry and Natural Sciences Department, 16561 Universitat Jaume I, Castellón 12071, Spain; ‡ Hochschule Geisenheim University, Department of Crop Protection, Von-Lade-Str. 1, Geisenheim 65366, Germany; § 9167 Bielefeld University, Department of Chemical Ecology, Universitätsstraße 25, Bielefeld 33615, Germany

**Keywords:** global warming, Planococcus ficus, Anagyrus vladimiri, honeydew, carbohydrates, metabolomics plant-derived food source, Vitis vinifera, nutritional ecology

## Abstract

Honeydew excreted by phloem-feeding insects is a major plant-derived carbohydrate resource in agroecosystems, yet its chemical sensitivity to climate change remains poorly understood. We examined how elevated atmospheric CO_2_ and water limitation affect honeydew primary metabolites and a honeydew-feeding parasitoid. Using a tritrophic system comprising grapevine (*Vitis vinifera*), the vine mealybug (*Planococcus ficus*), and its parasitoid (*Anagyrus vladimiri*), plants were grown under ambient or elevated CO_2_ and well-watered or water limitation conditions. Honeydew was quantified, primary metabolites were profiled by GC–MS, and parasitoid longevity and egg load were measured. Honeydew quantity was unaffected by treatments, but elevated CO_2_ reduced sugar concentrations, including those of sucrose and erlose, while water limitation increased raffinose and *myo*-inositol concentrations. These chemical changes were associated with reduced parasitoid longevity and fecundity, demonstrating that climate-driven alterations in honeydew composition may compromise its nutritional quality, with implications for biological control in vineyard systems.

## Graphical abstract

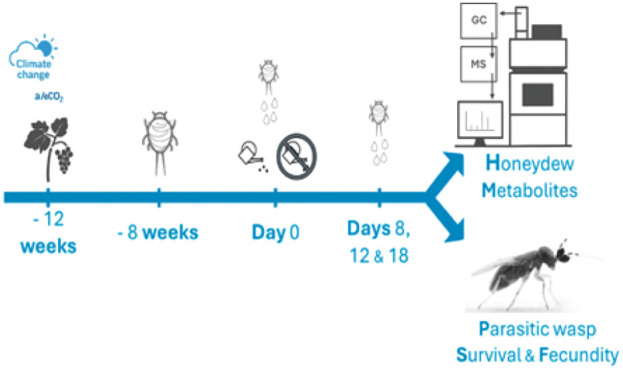

## 1 Introduction

Biological control agents are essential for sustainable pest management in agricultural systems.^1^ Most biological control agents are omnivorous, feeding on both pest organisms and plant-derived resources. In natural and managed ecosystems, key plant-based food sources include pollen, (extra)­floral nectar, plant guttation droplets, and honeydew—the sugar-rich excretion produced by phloem sap-feeding insects. Among these, honeydew stands out as one of the most abundant and readily accessible sugar and protein sources in agricultural landscapes.^2–5^ Honeydew is important in supporting beneficial insects, such as parasitic wasps and predators.^2^ Thus, managing honeydew availability in agroecosystems holds promise as a strategy to boost biological control agents populations and enhance biological control efforts.^6^ Vineyards are typically replanted every 30 years,^7^ they have the potential to serve as a long-term refuge for biological control agents. Grapevine (*Vitis vinifera* L., Vitaceae) is wind-pollinated and does not produce nectar.^8^ Hence, honeydew from phloem-feeding pests might be one of the most important sugar sources for biological control agents in vineyards, at least if other flowering plants are absent.

The vine mealybug, *Planococcus ficus* Signoret (Hemiptera: Pseudococcidae), is a phloem-feeding pest on grapevine in Mediterranean climate regions worldwide.^9^ The chemical composition of the honeydew produced by this species remains poorly understood, with only one study conducted over 50 years ago using now outdated techniques.^10^ Interestingly, honeydew of the closely related *Planococcus citri* Risso (Hemiptera: Pseudococcidae) improved fitness parameters of the parasitoid wasp *Anagyrus vladimiri* Triapitsyn (Hymenoptera: Encyrtidae).^4^ This wasp is one of the most prevalent and effective parasitoid species targeting mealybugs, including *P. ficus*, in vineyard agroecosystems.^11^

However, the availability and quality of honeydew are inherently variable and often dependent on environmental and seasonal factors.^3^ With ongoing climate change, a deeper understanding of its effects on honeydew traits is essential. Indeed, it has been highlighted that rising atmospheric CO_2_ levels and changing rainfall patterns add complexity to understanding and managing plant–insect interactions in agroecosystems.^12,13^ Several effects of elevated CO_2_ (eCO_2_) and water limitation on grapevine have been reported. For example, eCO_2_ is known to enhance the photosynthetic activity, carbon/nitrogen ratio, growth, and yield in grapevine.^14^ Water use efficiency of this plant species may be improved under eCO_2_ ^15^ or not, likely depending on the cultivar.^14^ Although the grapevine photosynthetic machinery is quite tolerant to mild and medium water deficit, photosynthetic rates decrease under severe or long-term drought which can have negative effects on yield.^16^ Grapevine plants accumulate osmolytes like proline and nonstructural carbohydrates in a cultivar-dependent manner.^16,17^ However, it remains underexplored how eCO_2_ and water limitation, alone and in combination, affect the honeydew produced by phloem-feeders, and if this impacts honeydew-feeding biological control agents.

By investigating honeydew as a crucial nutrient source that connects multiple trophic levels, this study aimed to elucidate how climate change-induced shifts in plants may cascade through tritrophic interactions involving the plant, a phloem-feeder, and a natural enemy. Thus, the questions addressed in this study were (i) whether and how eCO_2_ and water limitation alter the honeydew quantity and quality of *P. ficus* feeding on grapevine, i.e., honeydew fresh mass, dry matter content, density, and primary metabolites including sugars and amino acids, and (ii) whether these changes impact the honeydew nutritional value for *A. vladimiri* and, consequently, its fitness, as measured by longevity and fecundity.

## 2 Materials and Methods

### 2.1 Insects

#### 2.1.1 Mealybugs

*Planococcus ficus* were obtained from the Department of Crop Protection at Geisenheim University, Germany, and maintained on sprouted potatoes under controlled conditions [26 ± 1 °C (mean ± SD), 60 ± 12% relative humidity (RH), light/dark period: 16:8 h]. Potatoes were renewed regularly.

#### 2.1.2 Parasitic Wasps

The parasitoid *A. vladimiri* was obtained from Agrobio SL (Almería, Spain) in mummies of *Planococcus citri*. They were introduced in a plastic cage (47.5 cm × 47.5 cm × 47.5 cm; BugDorm-2; MegaView Science, Taiwan) and kept in the laboratory at room temperature until adults emerged. Unfed newly emerged parasitoids were collected daily and were used in the experiments.

### 2.2 Plant-Mediated Effects of eCO_2_ and Water Limitation on *Planococcus ficus*Honeydew

#### 2.2.1 Honeydew Sampling

The experiment was performed in a full-factorial design to test the effects of eCO_2_ and water limitation. Grapevine (*Vitis vinifera* L.“Johanniter”) was produced from cuttings and cultivated in pots in the greenhouse for 12 weeks until they reached a height of 60 cm, corresponding to grapevine developmental stage BBCH 9: nine leaves or more unfolded.^18^ Then, 32 plants were transferred into two Fitotron climate chambers (Fitotron Standard Growth Chamber SGC 120, Weiss Gallenkamp, Germany; 16 plants per chamber), where they were maintained under ambient (400 ppm, aCO_2_) or elevated (800 ppm, eCO_2_) CO_2_ concentrations, respectively, at a day-night cycle of 16:8 h, 24 °C:14 °C, and 60% RH. These CO_2_ concentrations were chosen, because current CO_2_ levels are approximately 400 ppm, future scenarios project this number to double or even triple by the year 2100.^19^ For the present study we chose an intermediate scenario with 800 ppm of CO_2_. Plants were watered twice per week. After 4 weeks, the plants were infested with mealybugs. First instar mealybug crawlers were collected by briefly placing detached grapevine leaves on the mealybug culture; leaves were then transferred to experimental plants. After eight more weeks, water limitation treatments started (defined as day 0), i.e., 6 plants per climate chamber were watered daily until field capacity (well-watered), and 10 plants per chamber were not watered. From this time point onward, the predawn leaf water potential (Ψ) was monitored every 2–5 days using a Scholander pressure chamber (Soilmoisture Equipment Corp., Santa Barbara, CA, USA), selecting fully developed leaves at comparable plant heights (ca. 40 cm); plants had between 5 and 15 leaves and were not flowering.

Honeydew was collected at three time points: just before the start of the water limitation treatment (day 0); when leaf Ψ showed significant differences between well-watered and not watered plants (day 8; GLM, *F*_1,31_ = 44.48, *p* < 0.001); and when plants exhibited clear signs of drought stress (e.g., wilting) on day 12 and 18 under eCO_2_ and aCO_2_, respectively. We followed the procedures published by Urbaneja-Bernat et al.^20^ with slight modifications described below. Honeydew was sampled from mealybugs on different leaves at each sampling event to avoid repeated measurements. For the quantification of honeydew and the chemical analyses, clean pieces of aluminum foil, held in place using clip cages (diameter 5 cm), were attached to one leaf per plant at locations where mealybugs of mixed developmental stages were present. The mealybugs present per clip cage were counted. After 48 h, the aluminum foils were removed and 1 μL of honeydew was immediately collected from each foil with a pipet (Research Plus, Eppendorf, Hamburg, Germany), diluted with 50 μL water (Millipore), flash-frozen in liquid nitrogen, and stored at −80 °C until lyophilization. The aluminum pieces were weighed before and after honeydew collection, after removal of 1 μL of honeydew, and after drying at 24 °C and 40% RH for 1 week to measure the fresh mass of the produced honeydew as well as its dry matter content (mass after drying/before drying, in percent) and its density (mass per volume) if possible.

The fresh mass of honeydew per aluminum piece was divided by the number of mealybugs present to calculate an approximate average amount produced per mealybug. Sample sizes per treatment vary (n = 14–34) due to the sampling schedule and due to inherent challenges in mealybug honeydew collection. For the feeding experiments with parasitoids, pieces of Parafilm (Heathrow Scientific, Vernon Hills, IL, USA) were placed below the above-described mealybug-infested grapevine plants to collect honeydew droplets, following the procedures published by Urbaneja-Bernat et al.^4^ After 48 h, the Parafilm pieces with honeydew were stored at −20 °C for ca. 10 months until feeding experiments started.

#### 2.2.2 Honeydew Primary Metabolites Analysis

Profiling of primary metabolites in the honeydew was done in n = 7–15 samples per treatment as previously described^21,22^ with some modifications. Lyophilized samples were extracted with 80% methanol (v:v; methanol: LC–MS grade, Fisher Scientific, Loughborough, UK) containing D-(−)-salicin (Roth, Karlsruhe, Germany) as internal standard. Subsamples of the extracts were dried under nitrogen gas at 37 °C, followed by a two-step derivatization at 37 °C: methoximation with *O*-methylhydroxylamine hydrochloride (Alfa Aesar, Kandel, Germany; Sigma-Aldrich, St. Louis, MO, USA) in a concentration of 20 mg mL^–1^ in pyridine (Fisher Scientific; Sigma-Aldrich, Steinheim, Germany) for 90 min, followed by silylation (*N*-methyl-*N*-trimethylsilyl-trifluoroacetamide; Macherey-Nagel, Düren, Germany) for 30 min. Samples were diluted with pyridine and analyzed by gas chromatography coupled to mass spectrometry (GC-2010 Plus, QP2020; Shimadzu, Kyoto, Japan). Analytes were injected at 225 °C at a split ratio of 1:10 and separated on a VF-5 ms column (Agilent Technologies, Waldbronn, Germany; 30 m × 0.25 mm × 0.25 μm, with guard column of ca. 8 m) using a column flow rate of the carrier gas (helium) of 1.14 mL min^–1^ and the following oven temperature program: 80 °C (held for 3 min), linear increase at 5 °C min^–1^ to 325 °C (held for 6 min). The transfer line (interface) was operated at 250 °C, the ion source at 230 °C. Electron impact positive ionization at 70 eV was applied. Detection was done in a mass-to-charge ratio (*m*/*z*) range of 40 to 600. Six blanks, i.e., samples without biological material, were processed and measured in between. A series of *n*-alkanes (C_7_–C_40_; Sigma-Aldrich) was analyzed to calculate arithmetic retention indices (AI).^23^

GC–MS Postrun Analysis 4.45 (Shimadzu) was used for data analysis. Identification of analytes was done by comparing their AI as well as mass spectra (including qualifier and quantifier *m*/*z*) with reference standards measured along with the samples, an in-house database, and the Golm Metabolome Database.^24,25^ All analytes (identified metabolites or nonidentified analytes) that did not, or occurred much less, in the blanks were quantified based on the peak heights of total ion count chromatograms. The peak heights were divided by those of the analyte belonging to the internal standard. Blank subtraction was done using average peak heights found in the blanks. Peak heights of analytes belonging to the same metabolite were summed up. Then, peak heights were divided by the honeydew volume (1 μL each). Only metabolites or nonidentified analytes that occurred in at least four samples were left in the data set. Analytes belonging to glutamic acid and glutamine were removed, as conversions between these metabolites and the formation of artifacts during silylation hamper their valid quantification.^26^

Plants can respond to drought with increasing phloem osmolality, e.g., through raffinose accumulation.^27^ Phloem sap feeders may practice osmotic adjustment when taking up phloem sap with a high osmotic pressure, as shown for aphids that convert sucrose to oligosaccharides.^28^ To assess whether mealybugs possess a similar capacity for osmoregulation, we calculated the oligosaccharide index as the percentage of oligosaccharides (trisaccharides: raffinose + erlose) per total sugar (sum of tri-, di-, and monosaccharides), and examined if this index varies with host plant water potential.

### 2.3 Honeydew-Mediated Effects of eCO_2_ and Water Limitation on Parasitoid Fitness

To investigate the effects of eCO_2_ and water limitation on the suitability of *P. ficus* honeydew as a food source for *A. vladimiri*, six different diets were tested using newly emerged female wasps. In addition to mealybug honeydew samples from the experiment described above [section 2.2; (i) aCO_2_ well-watered, (ii) eCO_2_ well-watered, (iii) aCO_2_ not watered, (iv) eCO_2_ not watered], (v) a negative control diet consisting of distilled water only as well as (vi) a 1 M sucrose solution with 7 μg mL^–1^ superoxide dismutase (SOD; Sigma-Aldrich, S9697-15KU) as positive control were offered to the parasitoids. SOD is a defense-related plant protein (antioxidant enzyme) present in the honeydew of phloem sap feeders, such as mealybugs, which was shown to have positive effects on the third trophic level.^4^ The concentrations of sucrose and SOD were selected based on previous studies.^4,29^

To assess parasitoid survival (longevity), newly emerged (<12 h old) unfed *A. vladimiri* females were placed individually per diet (n = 25) into glass vials (height 7 cm, diameter 1.2 cm), with the diet provided *ad libitum* (survival experiment). Water was supplied via cotton balls, which also served to seal the vials. The diets were offered on 1 cm^2^ Parafilm pieces, and both food and water sources were renewed daily. Survival was monitored daily until all individuals had died. To determine the effect of the diet on egg load, a separate experiment (egg load experiment) was performed. Newly emerged unfed males and females (<12 h) were placed inside a transparent, cylindrical plastic cup (946 mL; diameter 114 mm; height 127 mm; Paper Mart, CA, USA) and were provided one of the six diets described above *ad libitum* for 24 h to ensure mating. After 24 h (day 1), n = 15 females per diet were killed and stored at −20 °C. Another 30 females per diet were individually placed into glass vials similar to the ones used in the survival experiment, with the corresponding diet offered *ad libitum*. After 3 and 7 days, females (n = 15 per diet and day) were killed and stored at −20 °C. The egg load of the females was assessed under a stereomicroscope after placing the wasps in a drop of water. The ovaries were extracted from the abdomen by applying slight pressure on the thorax with pins. The ovaries were photographed and the number of mature eggs was recorded.

### 2.4 Statistical Analyses

All statistical analyses were conducted using R (versions 4.2.1 and 4.3.1, R Core Team, 2022) software with a significance threshold of 5% (α = 0.05). Box-whisker plots were prepared using the R package *ggplot2*. The predawn leaf Ψ at the time of honeydew sampling, fresh and dry mass of honeydew produced per mealybug, honeydew dry matter content, and the concentrations of single metabolites detected in honeydew were evaluated using generalized linear models (GLM; Gamma distribution, inverse link function; R package *stats*) with the factors CO_2_ (factor levels aCO_2_, eCO_2_), and H_2_O (well-watered, not watered), as well as their interaction. To all metabolite concentrations, 1 × 10^–9^ was added to avoid zeros in case of not detected metabolites, as required by Gamma distribution. Correlations between predawn leaf Ψ and oligosaccharide index were evaluated using Kendall’s correlation analyses (R package *stats*, correlation plot: R package *ggpubr*). To compare the primary metabolite profiles of the honeydew samples, a nonmetric multidimensional scaling (NMDS) analysis was conducted (R package *vegan*), applying Wisconsin double standardization of square root-transformed data and Kulczynski distances. To assess responses of individual metabolites to elevated CO_2_ and/or water limitation, fold changes were calculated, dividing mean concentrations in the treatment group (eCO_2_ well-watered or aCO_2_ not watered or eCO_2_ not watered) by the mean concentration in the common control group (aCO_2_, well-watered). To compare responses between treatments and metabolites, a cluster heatmap was constructed, using log_2_-scaled fold changes of these three pairwise comparisons, with average linkage hierarchical clustering both over treatment groups and metabolites, relying on Pearson correlation distance matrices. This was done in Cluster 3.0 ^30^ with visualization in JavaTreeView.^31^ The survival of *A. vladimiri* females kept on the six diets was plotted using Kaplan–Meier curves. These curves were compared between groups using overall and pairwise log-rank tests with Bonferroni correction (R packages *survival, survminer*). A GLM with a Poisson distribution and a log link function was applied to test for differences in the number of mature eggs per female kept on the different diets, separately for each time point (1, 3, and 7 days after emergence), followed by separation of the means using estimated marginal means and Bonferroni correction.

## 3 Results

### 3.1 Plant-Mediated Effects of eCO_2_ and Water Limitation on *P. ficus*Honeydew

The predawn leaf water potential Ψ at the time of honeydew sampling was significantly affected by the CO_2_ treatment (GLM: *F*_1,73_ = 8.23, *p =* 0.005) as well as by the H_2_O treatment (*F*_1,73_ = 26.38, *p* < 0.001), with no significant interaction between these factors (*F*_1,73_ = 0.10, *p =* 0.76). Plants subjected to water limitation showed significantly lower predawn leaf Ψ than well-watered plants under aCO_2_ and eCO_2_ conditions (Figure 1A). The lowest predawn leaf Ψ was found for plants grown under eCO_2_ and water limitation. Water limitation progressed faster under eCO_2_. On day 12 of water limitation, eCO_2_-plants already showed clear signs of wilting and very low predawn leaf Ψ, while for aCO_2_-plants this was only the case on day 18 (data not shown). The honeydew quantity produced per mealybug was not significantly affected by the CO_2_, the H_2_O treatment, or the interaction of the factors—neither fresh mass (CO_2_: *F*_1,95_ = 0.35, *p =* 0.56; H_2_O: *F*_1,95_ = 2.06, *p =* 0.16; CO_2_*H_2_O: *F*_1,95_ = 0.68, *p =* 0.41; Figure 1B), honeydew dry matter content (CO_2_: F_1,87_ = 0.07, *p =* 0.79; H_2_O: F_1,87_ = 1.86, *p =* 0.18; CO_2_*H_2_O: *F*_1,87_ = 0.40, *p =* 0.53; Figure 1C), nor honeydew density (CO_2_: *F*_1,58_ = 1.68, *p =* 0.20; H_2_O: F_1,58_ = 3.10, *p =* 0.08; CO_2_*H_2_O: F_1,58_ = 0.03, *p =* 0.87; n = 12–30; data not shown).

**Figure 1:**
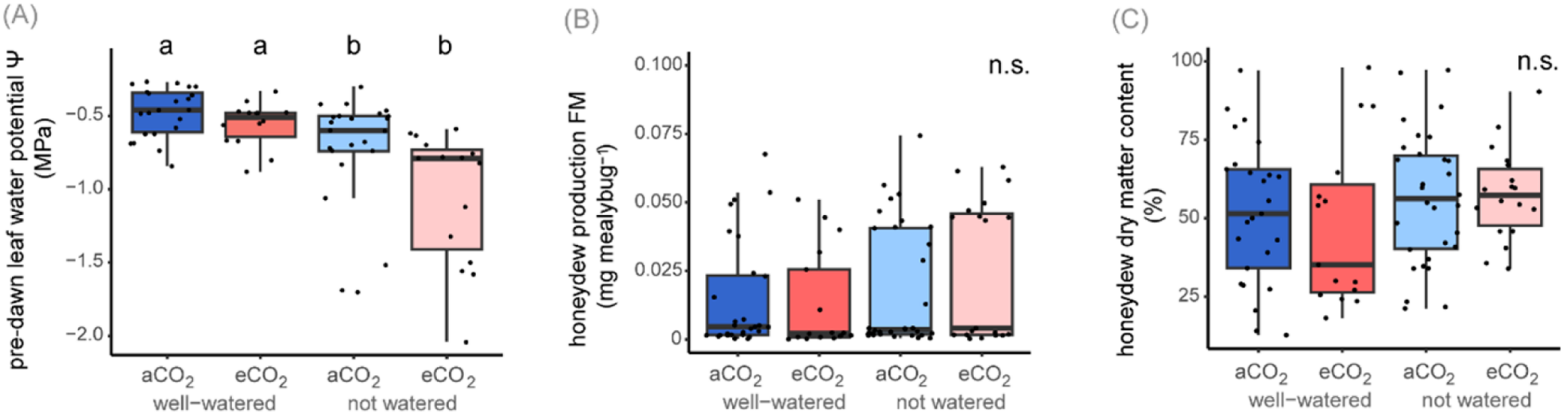
Water status of *Vitis vinifera* plants and quantity-related traits of mealybug (*Planococcus ficus*) honeydew produced on plants that were subjected to different CO_2_ (aCO_2_, ambient CO_2_; eCO_2_, elevated CO_2_) and H_2_O (well-watered, not watered) treatments. (A) Predawn leaf water potential Ψ (MPa) of plants when honeydew samples were collected for plants cultivated at eCO_2_ and aCO_2_, respectively, (B) honeydew production per mealybug (mg fresh mass; FM), (C) honeydew dry matter content in percent. Horizontal lines in the box-whisker plots mark the median while the lower and upper hinges correspond to the first and third quartiles (i.e., the 25th and 75th percentiles). The upper and lower whiskers extend to the largest and smallest value, respectively, but no further than 1.5 the interquartile range. Raw data points are shown as black circles. Different letters indicate significant differences between treatments (GLM, *n* = 14–34). n.s.: not significant.

In honeydew, we detected 42 metabolites, including unknown analytes. We could identify several sugars (monosaccharides: glucose, fructose, galactose, glucose-6-phosphate, fructose-6-phosphate; disaccharides: sucrose, α,α′-trehalose, maltose; oligosaccharides: erlose, raffinose), polyols (*myo*-inositol, galactinol, sorbitol), amino acids (valine, threonine, isoleucine, phenylalanine, glycine, serine, proline), other organic acids (malic acid, tartaric acid, octadecanoic acid, citric acid, dehydroascorbic acid dimer), and one inorganic acid (phosphoric acid), while 16 analytes could not be identified but are assumed to be primary metabolites as well (Supplementary Table S1). The metabolite profiles of the honeydew from mealybugs on host plants of the different treatment groups (CO_2_, H_2_O) largely overlapped, as assessed by nonmetric multidimensional scaling (Figure 2). Within the nonwatered plants, the honeydew samples from mealybugs on plants with the lowest predawn leaf Ψ appeared to be distinct. A cluster heatmap based on fold changes revealed that several metabolites in the mealybug honeydew responded to the CO_2_ and/or the H_2_O treatment of the host plants (Figure 3). Several amino acids and unknown analytes showed higher concentrations under eCO_2_, water limitation, as well as the combination of both, with the response to the combination (eCO_2_, not watered) being most pronounced. Several metabolites had slightly higher concentrations in both water limitation groups, while they were not or less affected by eCO_2_.

**Figure 2:**
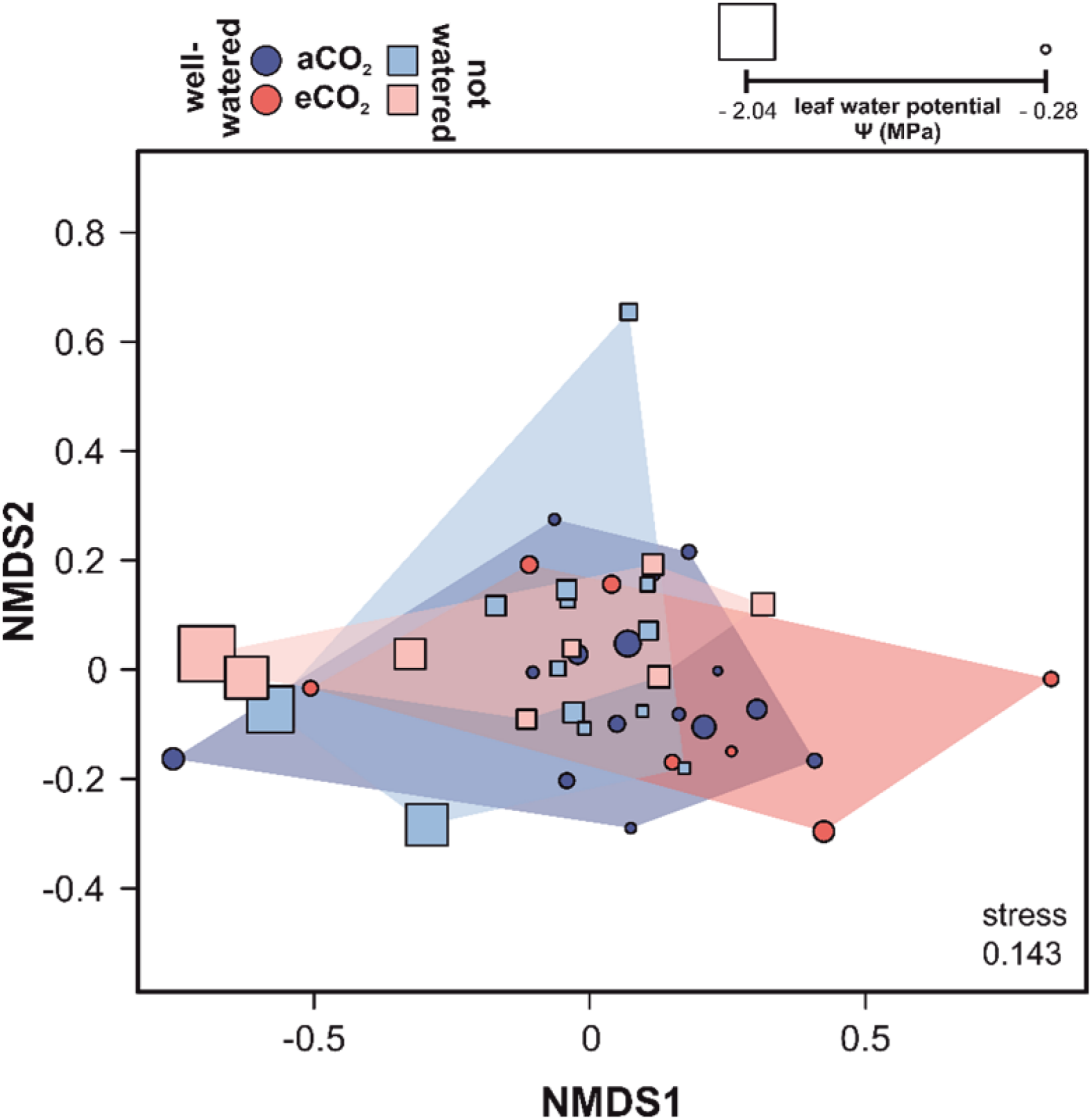
Nonmetric multidimensional scaling (NMDS) of 42 metabolites, including unknown analytes, in mealybug (*Planococcus ficus*) honeydew produced on *Vitis vinifera*plants subjected to different CO_2_ (aCO_2_, ambient CO_2_: blue; eCO_2_, elevated CO_2_: red) and H_2_O (well-watered: circles, dark colors; not watered: squares, light colors) treatments. The stress value of the NMDS analysis is indicated at the bottom right. The treatment groups are surrounded by convex hulls. The larger the size of the symbol, the more severe the water limitation of the host plant (i.e., lower predawn leaf water potential Ψ) (*n* = 7–15).

**Figure 3:**
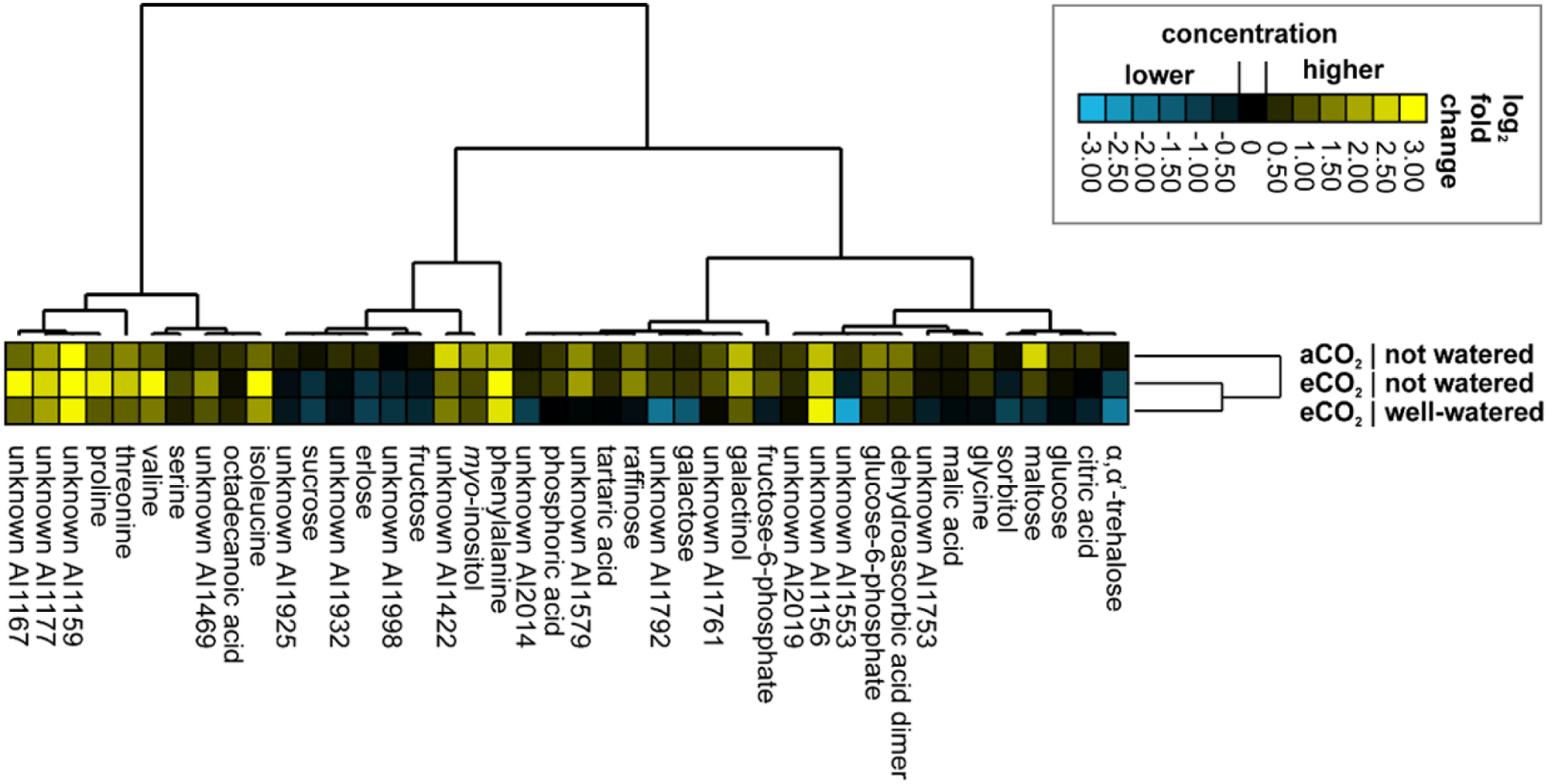
Cluster heatmap of 42 metabolites, including unknown analytes, in mealybug (*Planococcus ficus*) honeydew produced on *Vitis vinifera* plants subjected to different CO_2_ (aCO_2_, ambient CO_2_; eCO_2_, elevated CO_2_) and H_2_O (well-watered, not watered) treatments. In the heatmap, log_2_-scaled fold changes of metabolite concentrations (means of treatment groups with *n* = 7–15) in comparison to the common control group (aCO_2_, well-watered) are given. Fold changes for metabolites that showed higher concentrations in the indicated group compared to the common control group are indicated in yellow, while those with lower concentrations are indicated in blue.

Some sugars and unknown analytes showed slightly lower concentrations in both eCO_2_ groups, while some others had lower concentrations in the well-watered eCO_2_ group only (Figure 3). Some identified metabolites were significantly affected by the CO_2_ treatment or the H_2_O treatment, while none were affected by the interaction of both factors (Figure 4 and Table S2). The concentrations of sucrose, α,α′-trehalose, erlose, and sorbitol in the honeydew were lower when mealybug host plants were cultivated under eCO_2_ compared to aCO_2_ (Supplementary Tables S1,S2 and Figure 4A,B,C,E). Concentrations of raffinose, *myo*-inositol, and dehydroascorbic acid dimer in honeydew were higher when mealybugs fed on water-limited host plants compared to well-watered ones (Supplementary Tables S1,S2 and Figure 4D,F,G). With decreasing predawn leaf water potential (Ψ) of the host plant (indicating increasing water limitation), the oligosaccharide index significantly increased (R = −0.36, z = −3.34, *p* < 0.001, τ = −0.36; Figure 4 H).

**Figure 4:**
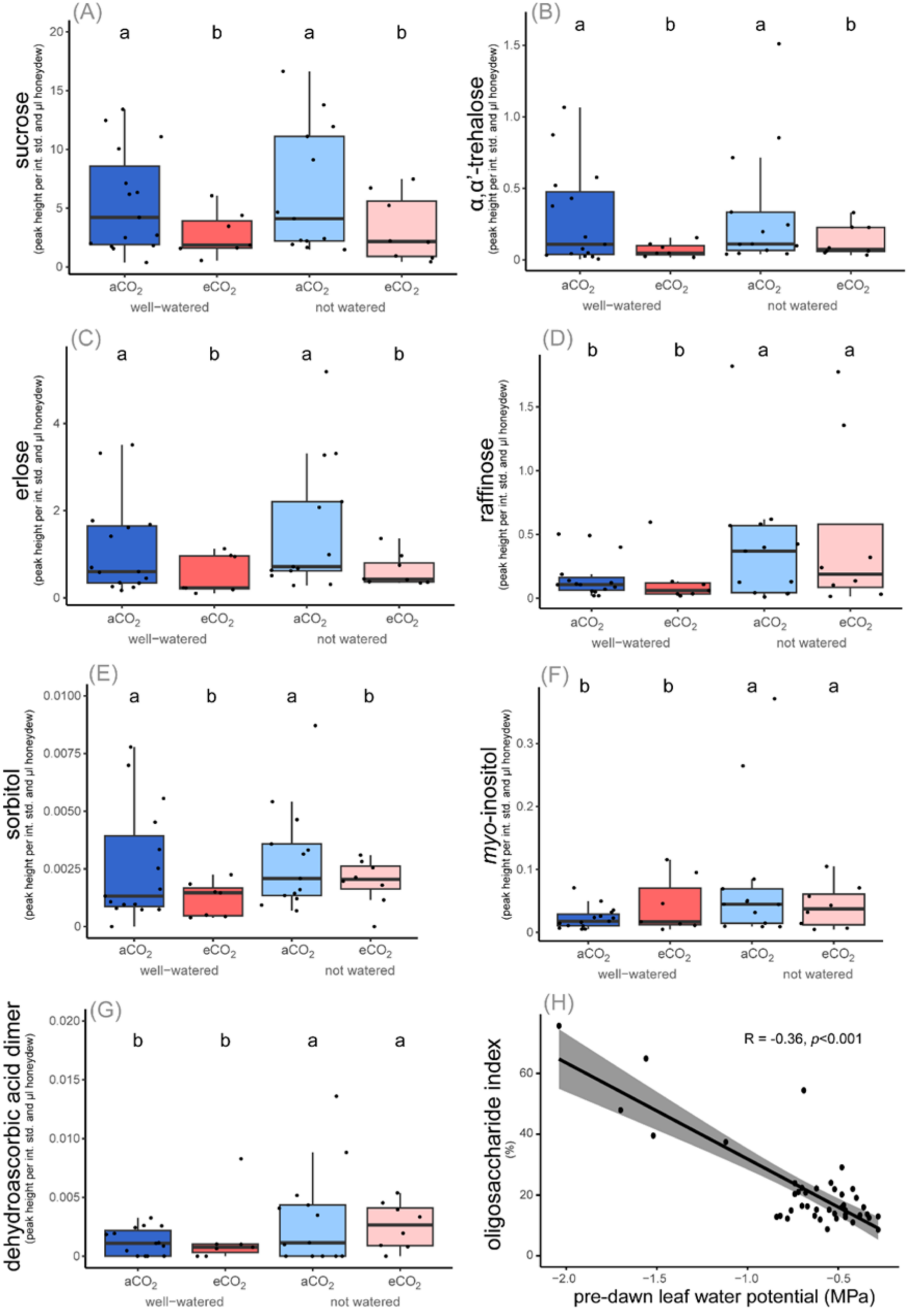
Concentrations of metabolites (A–G) in mealybug (*Planococcus ficus*) honeydew that were significantly affected by the growing conditions of their host plants (*Vitis vinifera*). These plants were subjected to different CO_2_ (aCO_2_, ambient CO_2_; eCO_2_, elevated CO_2_) and H_2_O (well-watered, not watered) treatments. Horizontal lines in the box-whisker plots mark the median, while the lower and upper hinges correspond to the first and third quartiles (i.e., the 25th and 75th percentiles). The upper and lower whiskers extend to the largest and smallest value, respectively, but no further than 1.5 times the interquartile range. Raw data points are shown as black circles. Different letters indicate significant differences between treatments (GLM followed by Tukey tests, *n* = 7–15). In (H), the relationship between the oligosaccharide index of the mealybug honeydew and the host plant predawn leaf water potential Ψ is shown, including the linear regression line and the 95% confidence region indicated in gray (Kendall correlation analysis) (*n* = 7–15).

### 3.2 Honeydew-Mediated Effects of eCO_2_ and Water Limitation on Parasitoid Fitness

The survival of *A. vladimiri* females was significantly affected by the diet (log-rank test: χ^2^ = 588.59, *p* < 0.001; Figure 5). On the negative control diet (water), the survival was significantly lower than on all other diets. Access to mealybug honeydew produced under eCO_2_ compared to aCO_2_ concentration, regardless of water limitation, led to a significantly lower longevity of *A. vladimiri* females. Moreover, the longevity of wasps fed on honeydew produced on grapevine cultivated under aCO_2_ well-watered conditions was comparable to that of wasps fed on the positive control diet (sugar + SOD).

**Figure 5:**
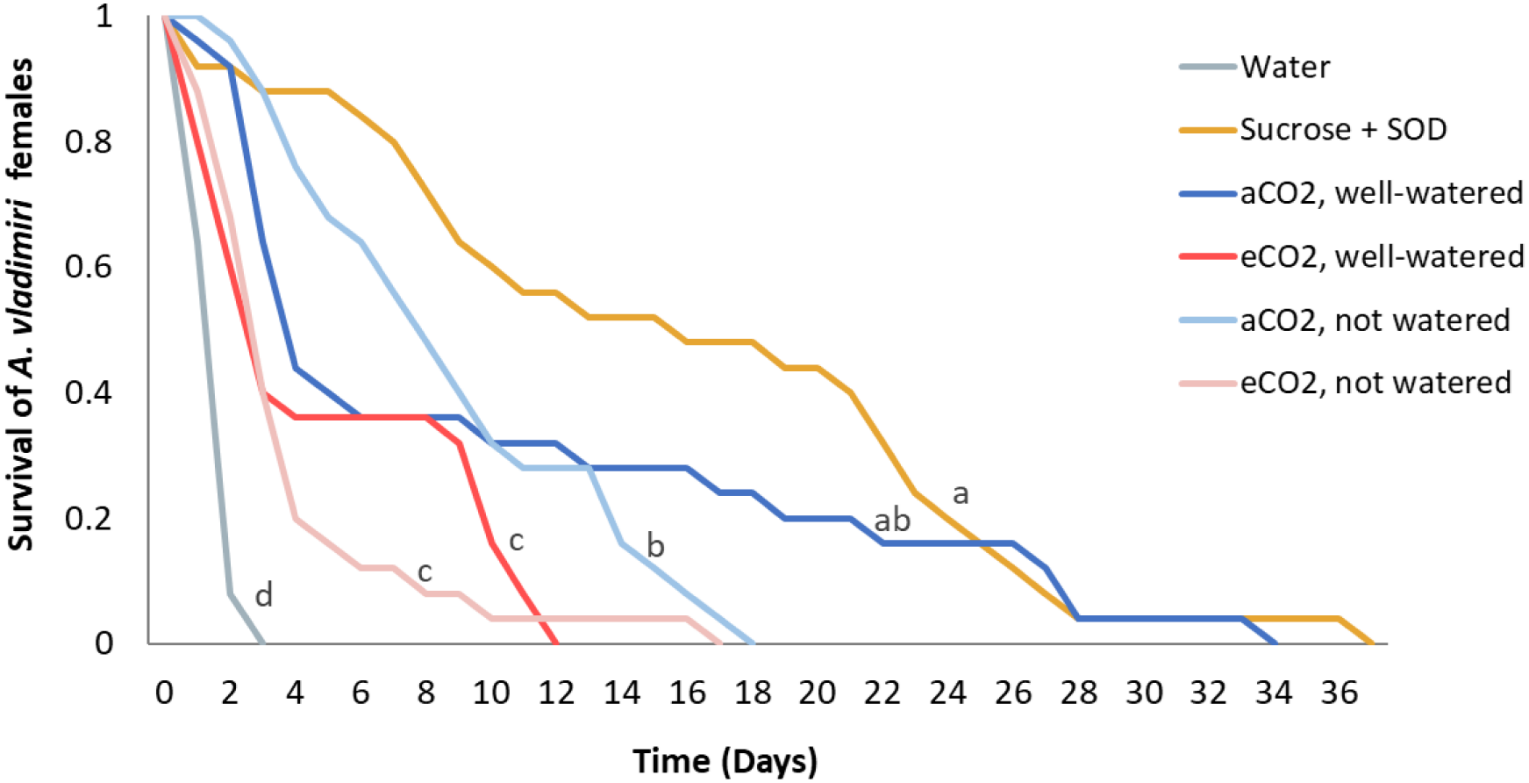
Survival curves [fraction of female parasitoids (*Anagyrus vladimiri*) alive] fed on six different diets: water, sucrose + superoxide dismutase (SOD), or honeydew collected from mealybugs (*Planococcus ficus*) on host plants (*Vitis vinifera*) subjected to different CO_2_ (aCO_2_, ambient CO_2_; eCO_2_, elevated CO_2_) and H_2_O (well-watered, not watered) treatments. Different letters indicate significant differences between treatments (log-rank tests with Bonferroni correction) (*n* = 25).

The egg load of newly emerged, unfed *A. vladimiri* females (<12 h old) was 4.4 ± 0.3 (mean ± SE) eggs per female (Figure 6). One day after emergence, the egg load of females differed significantly depending on the diet (GLM: χ^2^ = 59.86, *p* < 0.001), with those fed on any honeydew diet or the positive control (sucrose + SOD) having a higher egg load than females that had only access to water. At the two later time points (3- and 7-days post emergence), all females with only access to water and those with access to honeydew produced under eCO_2_ had already died. For the remaining females in the other diet groups, the egg load significantly differed between dietary treatments both at 3 days (χ^2^ = 66.92, *p* < 0.001) and at 7 days (χ^2^ = 21.31, *p* < 0.001) after emergence. At both time points, the egg load of wasps fed on honeydew produced under aCO_2_ and well-watered conditions was similar to the egg load of females fed on the sucrose + SOD diet. However, the egg load was significantly reduced in females fed on honeydew produced under aCO_2_ with water limitation.

**Figure 6:**
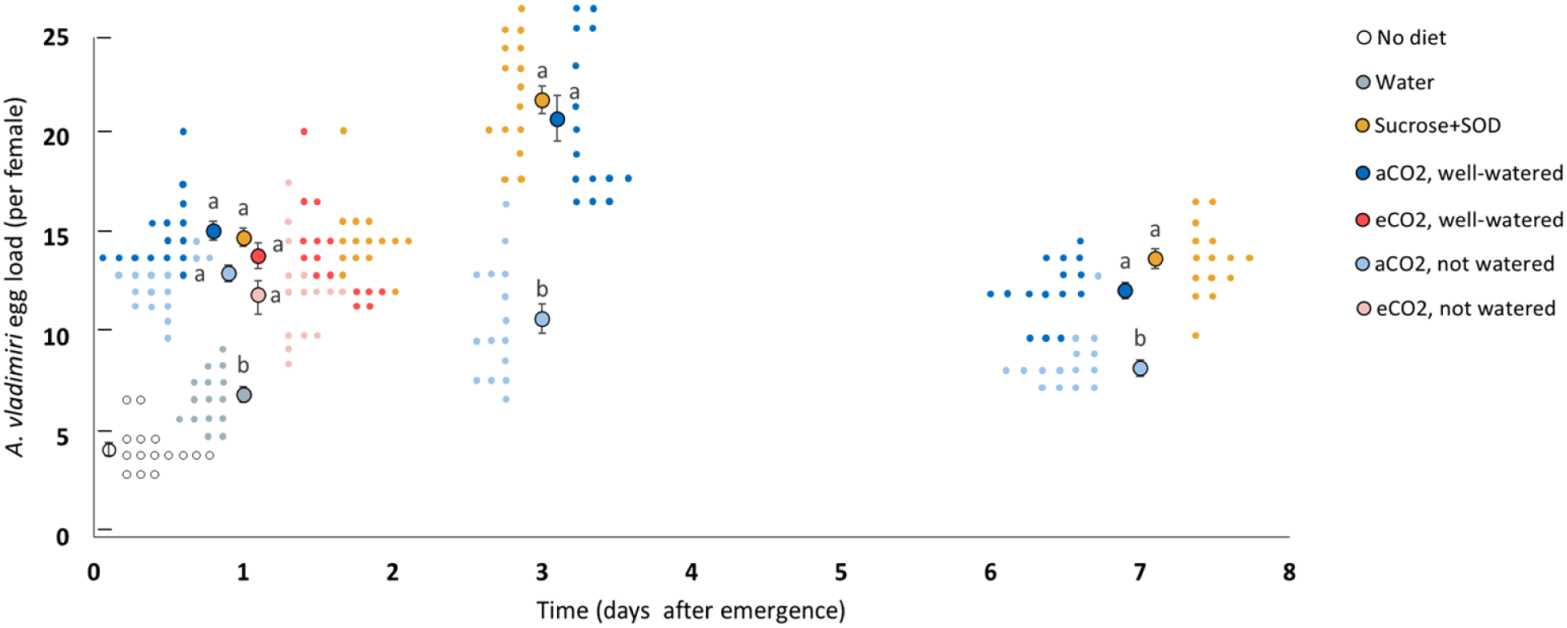
Egg load of newly emerged female parasitoids (*Anagyrus vladimiri*) under different dietary treatments. Six diets were offered for one, three, or 7 days: water, sucrose + superoxide dismutase (SOD), or honeydew collected from mealybugs (*Planococcus ficus*) on host plants (*Vitis vinifera*) subjected to different CO_2_ (aCO_2_, ambient CO_2_; eCO_2_, elevated CO_2_) and H_2_O (well-watered, not watered) treatments (*n* = 15). Circles represent individual females; larger circles and error bars show means ± SE. Treatment groups were statistically compared within time points, with different letters indicating significant difference between groups (Bonferroni-corrected tests). At day 3 and 7, all females of the treatment groups that are not shown had already died, while all (*n* = 15) females of the groups that are shown were still alive.

## 4 Discussion

Our study provides insights into the chemical composition of *P. ficus* honeydew produced on grapevine and how eCO_2_ and water limitation modify this composition, with consequent effects on the fitness of the parasitoid *A. vladimiri*. We found that while eCO_2_ intensified water limitation in grapevine plants, the honeydew quantity remained unaffected by eCO_2_ or water limitation. However, the concentrations of several primary metabolites in the honeydew were significantly altered by these factors, including sucrose, α,α′-trehalose, erlose, raffinose, *myo*-inositol, and sorbitol. Moreover, the survival and egg load of *A. vladimiri* differed among honeydew diets. These findings indicate that climate change-driven shifts in honeydew composition reduce the quality of this food source for biological control agents, presenting challenges for pest management strategies in vineyard systems.

The lower predawn leaf Ψ in not-watered grapevine plants indicates that the plants were not able to fully re-equilibrate with soil moisture overnight, reflecting sustained water deficit conditions and limited soil water availability. Interestingly, predawn leaf Ψ dropped faster in plants cultivated under elevated compared to ambient CO_2_ concentrations. This may be explained by increased or sustained stomatal conductance under eCO_2_, a response that has been previously observed in grapevine and other woody plants,^14,32^ which may have led to higher transpiration and thus a higher water loss by the plants. However, neither eCO_2_ nor water limitation affected honeydew production in terms of fresh^14,32^ or dry mass, dry matter content, or density. Honeydew excretion patterns are not well studied, but excretion frequencies have been shown to vary within and between individuals of the closely related mealybug species *Planococcus citri*.^33^ In line with our study, Blanchard et al.^34^ did not find any adjustments in aphid honeydew dry mass under different CO_2_ and temperature regimes. However, several studies report lower or higher amounts of aphid honeydew excreted under eCO_2_ ^35,36^ and water limitation. While there is no literature on plant water stress effects on mealybug honeydew production, the brown planthopper *Nilaparvata lugens* produces less honeydew on water-logged rice plants.^37^ Similarly, aphids reduced honeydew production when feeding on mildly and highly water-limited wheat, likely because they ingested less phloem sap per time compared to well-watered plants.^38^ We cannot rule out that the lack of an effect on honeydew quantity in our study might be attributed to our sampling method, which included mixed developmental stages of mealybugs that may have variable feeding rates or honeydew excretion rates. Future studies may benefit from standardizing the developmental stages of mealybugs to better capture potential changes in honeydew quantity.

Compared to the earlier study on honeydew composition of *P. ficus* fed on potato,^10^ which reported the presence of sucrose, glucose, fructose, and raffinose, our analysis identified additional sugars, including galactose, glucose-6-phosphate, maltose, α,α′-trehalose, and erlose in the honeydew of this insect species when fed on *V. vinifera*. Saleh and Salama^10^ did not detect any polyols; in contrast, we found sorbitol and *myo*-inositol. While they are known to accumulate in plants under drought stress,^27,39^ this discrepancy in metabolite occurrence between studies may result from differences in host plant species (potato vs grapevine) and/or methodological sensitivity. Most of the compounds we found in *P. ficus* honeydew have also been reported from grapevine tissues, and are thus likely plant phloem sap-derived. For instance, sucrose, which showed the highest abundance across our honeydew samples, is among the most abundant sugars in grapevine phloem exudates.^40^ Galactose, fructose, glucose (and their respective 6-phosphates), maltose, as well as raffinose, have been reported in grapevine petioles or phloem exudates.^17^ In contrast, the presence of erlose, which has not been reported from grapevine, suggests biosynthesis by *P. ficus* or its endosymbionts. Indeed, erlose is considered a ”signature sugar“ of hemipteran honeydew.^41^ The detected polyols, organic and the inorganic acid have been reported in grapevine.^42^ Octadecanoid acid might (at least partly) be an insect-derived artefact since it does occur in mealybug wax, e.g., of the giant mealybug *Drosicha stebbingii*.^43^

Interestingly, while certain metabolites like raffinose and *myo*-inositol showed higher concentrations in the honeydew of *P. ficus* fed on plants under water limitation, others like sucrose, α,α′-trehalose, and erlose were negatively influenced by elevated CO_2_. This nonuniform impact of environmental factors on metabolites highlights the complex biochemical responses involved, possibly due to species-specific mechanisms in grapevines that influence sugar concentrations in response to stress.^44^ Remarkably, sucrose, α,α′-trehalose, and erlose concentrations were lower in honeydew produced under eCO_2_, when grapevine photosynthesis is usually enhanced^14^ and sugar concentrations in plants are typically higher.^45^ However, whether elevated CO_2_ concentrations actually result in increased sugar concentrations in grapevine phloem sap is unknown to date and should be investigated in future studies. Once taken up by the mealybug, the composition of the phloem sap may have been markedly altered by the mealybugs and/or their endosymbionts, resulting in a honeydew sugar profile distinct from phloem sap. The endosymbiont composition of mealybugs can also change due to environmental conditions.^46^ However, the metabolic activity of mealybugs and their endosymbionts has, to our knowledge, not been studied to date and should be addressed in the future.

Honeydew produced on water-limited plants contained more oligosaccharides than that produced on well-watered plants (oligosaccharide index, Figure 4H). This may indicate that the plant phloem sap of water-limited plants was osmotically challenging for the mealybugs. To reduce the osmotic pressure, mealybugs may have been converting mono- and di- into oligosaccharides, as known from aphids,^28^ which may be referred to as niche conformance.^47^ Furthermore, we cannot exclude the possibility that we were only seeing part of the picture, i.e., that oligosaccharides larger than trisaccharides have been synthesized by grapevine, mealybugs and/or their endosymbionts, which would not have been detected by our GC–MS method.

The composition of mealybug honeydew significantly influenced the survival and egg load of *A. vladimiri* females. Notably, honeydew produced by mealybugs on plants under eCO_2_ reduced the survival of these parasitoids. Under aCO_2_, survival was high enough to monitor egg load for up to 7 days, showing negative effects of water limitation. These findings indicate that the nutritional profile of honeydew directly affects parasitoid fitness and align with prior research suggesting that honeydew quality plays a critical role for parasitoid energy reserves and fitness and thus for effective pest control.^2,48^ In line with this, honeydew exposed to 7 and 14 days under semifield conditions has been shown to improve *A. vladimiri* survival while reducing egg load, suggesting that changes in honeydew quality can have trait-specific consequences for natural enemies.^49^ Sugars, which constitute the majority of honeydew dry matter and originate from both plant and insect metabolism, are particularly important because the parasitoids rely on them as their primary energy source, with strong implications for their fitness-related traits.^2^

As mealybugs affect plants in a density-dependent manner and eCO_2_ has been shown to slightly increase the survival of *P. ficus* ^50,51^ different colony sizes may have had a plant-mediated feedback on the honeydew. We did not monitor *P. ficus* colony sizes, but infestation levels were high on all plants. Indeed, herbivory has been shown to alter honeydew composition. For example, defense-related plant proteins induced by herbivory, such as SOD, were present in the honeydew of seven phloem sap-feeding insect species, including *P. citri*.^4^ The common presence of SOD in honeydew may further contribute to the fitness of *A. vladimiri*, emphasizing the intricate dynamics of interactions across the trophic cascade. The increase in egg load after emergence indicates that *A. vladimiri* is a synovigenic parasitoid, i.e., continues to produce and mature eggs during adult life, as reported before.^4^ The parasitoid likely benefits from the proteins present in honeydew, which may be used for egg maturation.

Our findings suggest that climate change may pose challenges regarding the biological control of *A. vladimiri* in vineyard agroecosystems. This is particularly relevant given that perennial and wind-pollinated grapevine lack floral nectar sources, leaving honeydew as the primary sugar source for biological control agents. A decline in biological control agent survival and fitness due to altered honeydew quality may reduce their populations over time, potentially compromising pest control. Although we did not directly measure parasitism under field conditions, the observed effects on longevity and egg load suggest that climate change-driven effects on honeydew quality influence key parasitoid performance traits. These effects may also apply to other crop systems, such as citrus, where *A. vladimiri* parasitizes *P. citri*, although this remains hypothetical and depends on how citrus plants respond to climate change variables. This highlights the need for further research on alternative strategies to mitigate the effects of climate change on biological control agents, such as integrating flowering cover crops in vineyards and studying the effect of plant guttation to supplement sugar sources.^5^ To optimize biological control strategies for vineyard mealybugs, future research must evaluate the performance of parasitic wasps, such as parasitism rates, sex ratios, or behavioral responses, under climate change conditions. Our study emphasizes the importance of considering honeydew quality in integrated pest management strategies that support biological control agent populations and sustain biological control in agroecosystems under climate change.

## Supplementary Material



## Data Availability

Data are made available upon reasonable request.

## Notes

Metabolites and unknown analytes, along with their concentrations, in honeydew of the different treatment groups (Supplementary Table 1); test statistics (GLM) for metabolites (Supplementary Table 2) (PDF)

## Abbreviations

aCO_2_: ambient carbon dioxide concentration
AI: arithmetic retention index
eCO_2_: elevated carbon dioxide concentration
CO_2_: carbon dioxide
GC–MS: gas chromatography–mass spectrometry
GLM: general linear model
H_2_O: water
LC–MS: liquid chromatography–mass spectrometry
*m*/*z*: mass-to-charge ratio
NMDS: nonmetric multidimensional scaling
RH: relative humidity
SD: standard deviation
SE: standard error
SOD: superoxide dismutase
