# Supplementary material for: Elevated CO2 and Water Limitation Alter the Primary Metabolite Composition of Mealybug Honeydew, Reducing Parasitoid Fitness
Source: J Agric Food Chem. 2026 Jun 26;74(26):20261–71. doi: 10.1021/acs.jafc.5c17227 (PMC13352634; doi:10.1021/acs.jafc.5c17227)
Supplement: Supplementary file 1 [file jf5c17227_si_001.pdf]

Supporting Information

Elevated CO_2 and Water Limitation Alter the Primary Metabolite Composition of Mealybug Honeydew, Reducing Parasitoid Fitness

Pablo Urbaneja-Bernat[1*], Maria Schulze-Sylvester[2], Angeliki Syropoulou[1], Caroline Müller[3], Rabea Schweiger[3], Christine Becker[2]

[1] Plant Immunity and Biochemistry Group, Biology, Biochemistry and Natural Sciences Department, Universitat Jaume I, 12071, Castellón, Spain

[2] Hochschule Geisenheim University, Department of Crop Protection, Von-Lade-Str. 1, 65366 Geisenheim, Germany

[3] Bielefeld University, Department of Chemical Ecology, Universitätsstraße 25, 33615 Bielefeld, Germany

[*]Corresponding Author:

Pablo Urbaneja-Bernat

Av. Vicent Sos Baynat, s/n 12071 Castelló de la Plana, Espanya

Phone: +34 637 068 357

Email: pablo.urbaneja@uji.es

Supplementary Tables:

Table S1. Metabolites and unknown analytes detected in honeydew of mealybugs (*Planococcus ficus*) on grapevine (*Vitis vinifera*) plants subjected to different CO_2 (aCO_2, ambient CO_2; eCO_2, elevated CO_2) and H_2O (well-watered, not watered) treatments…………………………………… 3

Table S2. Test statistics for metabolites detected in mealybug (*Planococcus ficus*) honeydew produced on grapevine (*Vitis vinifera*) plants subjected to different CO_2 (aCO_2, ambient CO_2; eCO_2, elevated CO_2) and water (well-watered, not watered) treatments……………………….……. 5

Table S1: Metabolites and unknown analytes detected in honeydew of mealybugs (*Planococcus ficus*) on grapevine (*Vitis vinifera*) plants subjected to different CO_2 (aCO_2, ambient CO_2; eCO_2, elevated CO_2) and H_2O (well-watered, not watered) treatments. The names of the metabolites as well as the arithmetic retention indices (AI) of the corresponding analytes are given, with some metabolites being represented by more than one analyte. The metabolite concentrations in each treatment group are shown as means ± SD (n = 7-15).

**Metabolite**	**Arithmetic retention index**	**Concentrations** (peak heights per peak height of internal standard and µL honeydew)				
		**aCO_2**	**eCO_2**		**aCO_2**	**eCO_2**
		**well-watered**			**not watered**	
**Sugars**						
fructose	1859 | 1869	0.5169 ± 0.4036	0.3283 ± 0.2243		0.6133 ± 0.3407	0.3948 ± 0.2781
galactose	1877 | 1899	0.0030 ± 0.0060	0.0011 ± 0.0016		0.0043 ± 0.0051	0.0048 ± 0.0054
glucose	1883 | 1902	0.0597 ± 0.0744	0.0536 ± 0.0475		0.0954 ± 0.0975	0.0669 ± 0.0665
fructose-6-phosphate	2288	0.0003 ± 0.0004	0.0002 ± 0.0007		0.0005 ± 0.0015	0.0007 ± 0.0007
glucose-6-phosphate	2300	0.0003 ± 0.0004	0.0004 ± 0.0008		0.0009 ± 0.0023	0.0007 ± 0.0013
sucrose	2612	5.5658 ± 4.3669	2.7840 ± 1.9348		6.4015 ± 5.3654	3.2336 ± 2.8192
maltose	2711 | 2738	0.0012 ± 0.0042	0.0007 ± 0.0019		0.0072 ± 0.0188	0.0022 ± 0.0043
α,α'-trehalose	2718	0.2909 ± 0.3387	0.0696 ± 0.0503		0.3356 ± 0.4384	0.1341 ± 0.1109
raffinose	3347	0.1647 ± 0.1629	0.1408 ± 0.2052		0.3973 ± 0.4841	0.4969 ± 0.6759
erlose	3385	1.1327 ± 1.0831	0.5410 ± 0.4495		1.5985 ± 1.5199	0.6246 ± 0.3734
**Polyols**						
sorbitol	1920	0.0026 ± 0.0025	0.0012 ± 0.0008		0.0029 ± 0.0023	0.0019 ± 0.0010
myo-inositol	2075	0.0231 ± 0.0181	0.0433 ± 0.0447		0.0810 ± 0.1099	0.0416 ± 0.0349
galactinol	2958	0.0028 ± 0.0072	0.0060 ± 0.0157		0.0127 ± 0.0250	0.0128 ± 0.0209
**Amino acids**						
valine	1210	0.0030 ± 0.0105	0.0081 ± 0.0195		0.0067 ± 0.0174	0.0230 ± 0.0324
serine	1254 | 1354	0.0045 ± 0.0071	0.0058 ± 0.0113		0.0053 ± 0.0065	0.0080 ± 0.0087
isoleucine	1287	0.0015 ± 0.0050	0.0053 ± 0.0135		0.0035 ± 0.0085	0.0142 ± 0.0197
threonine	1290 | 1379	0.0032 ± 0.0098	0.0071 ± 0.0172		0.0095 ± 0.0197	0.0165 ± 0.0256
proline	1292	0.0017 ± 0.0065	0.0035 ± 0.0092		0.0039 ± 0.0132	0.0117 ± 0.0193
glycine	1300	0.0023 ± 0.0023	0.0020 ± 0.0024		0.0044 ± 0.0066	0.0036 ± 0.0025
phenylalanine	1548 | 1621	0.0008 ± 0.0022	0.0052 ± 0.0132		0.0037 ± 0.0102	0.0065 ± 0.0102
**Organic acids**						
malic acid	1483	0.0143 ± 0.0096	0.0128 ± 0.0128		0.0177 ± 0.0178	0.0165 ± 0.0092
tartaric acid	1631	0.0041 ± 0.0031	0.0040 ± 0.0059		0.0057 ± 0.0065	0.0060 ± 0.0049

citric acid	1810	0.0040 ± 0.0035	0.0027 ± 0.0038		0.0063 ± 0.0062	0.0039 ± 0.0018
dehydroascorbic acid dimer	1839	0.0012 ± 0.0011	0.0017 ± 0.0029		0.0032 ± 0.0041	0.0026 ± 0.0020
octadecanoic acid	2241	0.0076 ± 0.0060	0.0107 ± 0.0176		0.0117 ± 0.0146	0.0085 ± 0.0140
**Inorganic acid**						
phosphoric acid	1265	0.0173 ± 0.0117	0.0170 ± 0.0219		0.0272 ± 0.0221	0.0294 ± 0.0198
**Unknown analytes**						
unknown AI1156	1156	0.0004 ± 0.0014	0.0026 ± 0.0069		0.0017 ± 0.0041	0.0020 ± 0.0040
unknown AI1159	1159	0.0001 ± 0.0002	0.0004 ± 0.0004		0.0004 ± 0.0011	0.0014 ± 0.0021
unknown AI1167	1167	0.0002 ± 0.0004	0.0005 ± 0.0008		0.0005 ± 0.0004	0.0016 ± 0.0016
unknown AI1177	1177	0.0005 ± 0.0018	0.0018 ± 0.0046		0.0018 ± 0.0047	0.0027 ± 0.0051
unknown AI1422	1422	0.0006 ± 0.0014	0.0017 ± 0.0043		0.0035 ± 0.0068	0.0015 ± 0.0018
unknown AI1469	1469	0.0002 ± 0.0003	0.0004 ± 0.0008		0.0003 ± 0.0006	0.0006 ± 0.0013
unknown AI1553	1553	0.0010 ± 0.0011	0.0002 ± 0.0004		0.0016 ± 0.0019	0.0007 ± 0.0009
unknown AI1579	1579	0.0004 ± 0.0011	0.0004 ± 0.0007		0.0012 ± 0.0025	0.0013 ± 0.0026
unknown AI1753	1753	0.0019 ± 0.0009	0.0013 ± 0.0011		0.0025 ± 0.0013	0.0023 ± 0.0013
unknown AI1761	1761	0.0006 ± 0.0005	0.0006 ± 0.0006		0.0011 ± 0.0006	0.0011 ± 0.0006
unknown AI1792	1792	0.0002 ± 0.0006	0.0001 ± 0.0001		0.0004 ± 0.0005	0.0004 ± 0.0008
unknown AI1925	1925	0.0050 ± 0.0039	0.0039 ± 0.0024		0.0071 ± 0.0062	0.0043 ± 0.0029
unknown AI1932	1932	0.0038 ± 0.0028	0.0032 ± 0.0026		0.0055 ± 0.0042	0.0034 ± 0.0017
unknown AI1998	1998	0.0158 ± 0.0138	0.0090 ± 0.0063		0.0154 ± 0.0122	0.0105 ± 0.0067
unknown AI2014	2014	0.0007 ± 0.0006	0.0003 ± 0.0007		0.0009 ± 0.0012	0.0010 ± 0.0008
unknown AI2019	2019	0.0007 ± 0.0011	0.0007 ± 0.0009		0.0011 ± 0.0014	0.0009 ± 0.0012

Table S2. Test statistics for metabolites detected in mealybug (*Planococcus ficus*) honeydew produced on grapevine (*Vitis vinifera*) plants subjected to different CO_2 (aCO_2, ambient CO_2;

eCO_2, elevated CO_2) and water (well-watered, not watered) treatments. Only identified metabolites that were significantly affected by at least one of the factors CO_2, H_2O, or their interaction are shown. Results are based on generalized linear models; significant *p*-values are highlighted in bold. n = 7-15.

**Metabolite**	**Treatment factor**	**Degrees of freedom**	***F*-value**	***p*-value**
sucrose	CO_2	1,42	6.37	**0.016**
	H_2O	1,42	0.31	0.58
	CO_2 * H_2O	1,42	0.03	0.86
α, α'-trehalose	CO_2	1,42	8.38	**0.006**
	H_2O	1,42	0.35	0.56
	CO_2 * H_2O	1,42	1.05	0.31
erlose	CO_2	1,42	7.88	**0.008**
	H_2O	1,42	1.17	0.29
	CO_2 * H_2O	1,42	0.0001	0.99
raffinose	CO_2	1,42	0.25	0.62
	H_2O	1,42	7.19	**0.011**
	CO_2 * H_2O	1,42	0.16	0.69
sorbitol	CO_2	1,42	4.39	**0.043**
	H_2O	1,42	0.67	0.41
	CO_2 * H_2O	1,42	0.86	0.36
myo-inositol	CO_2	1,42	0.24	0.63
	H_2O	1,42	6.44	**0.015**
	CO_2 * H_2O	1,42	3.38	0.07
dehydroascorbic acid dimer	CO_2	1,42	0.002	0.96
	H_2O	1,42	4.58	**0.039**
	CO_2 * H_2O	1,42	0.50	0.49
